# 1064 nm Nd:YAG versus 940 nm diode laser-assisted periodontal therapy in stage II periodontitis: a randomized controlled trial

**DOI:** 10.1186/s12903-026-08676-x

**Published:** 2026-06-01

**Authors:** Nada W. Shaban, Gehan S. Kotry, Maha R. Taalab, Marwa A. Meheissen, Kinga Grzech-Leśniak

**Affiliations:** 1 Department of Periodontology, Faculty of Dentistry, Alexandria University, Alexandria, Egypt; 2 Department of Oral Medicine, Periodontology, Oral Diagnosis and Oral Radiology, Faculty of Dentistry, Alexandria University, Champolion St. Azarita, Alexandria, 21521 Egypt; 3 Department of Medical Microbiology & Immunology, Faculty of Medicine, Alexandria University, Alexandria, Egypt; 4 Department of Integrated Dentistry, Faculty of Medicine and Dentistry, Wrocław Medical University, Wrocław, Poland; 5 Department of Periodontics, School of Dentistry, Virginia Commonwealth University, Richmond, VA USA

**Keywords:** Periodontitis, Laser-assisted periodontal therapy, LANAP^®^, Nd:YAG 1064 nm, Diode 940 nm, *Treponema denticola, *and *Prevotella *intermedia

## Abstract

**Background:**

Stage II periodontitis develops in the presence of a dysbiotic subgingival biofilm that sustains chronic inflammation and progressive loss of periodontal support. While scaling and root planing (SRP) remains the primary treatment for periodontitis, adjunctive methods have been suggested to improve subgingival debridement and microbial control. Laser-assisted new attachment procedure (LANAP^®^) is a proprietary, minimally invasive protocol originally designed for the 1064 nm neodymium: yttrium–aluminum garnet (Nd:YAG) laser system. 940 nm diode laser preferentially interacts with inflamed, blood-rich tissues and pigmented periodontopathogens. This study aimed to evaluate the immediate microbiological and short-term clinical effects of laser-assisted periodontal therapy performed in accordance with the clinical principles of LANAP^®^, but without using the proprietary LANAP^®^ system.

**Methods:**

This randomized controlled trial included thirty-three stage II periodontitis patients, randomly allocated to three equal groups. Test group I received Nd:YAG 1064 nm laser-assisted therapy based on LANAP^®^-derived clinical steps; test group II received 940 nm diode laser-assisted periodontal therapy using a comparable, minimally invasive protocol; and control group III received SRP only. Subgingival biofilm samples were obtained at baseline and immediately following each intervention. *T. denticola* and *P. intermedia* levels were measured using real-time quantitative polymerase chain reaction (qPCR). Clinical outcomes, including clinical attachment level (CAL), probing pocket depth (PPD), and bleeding on probing (BOP), were documented at baseline and at 3-month follow-up.

**Results:**

All three treatment modalities significantly reduced *T. denticola* and *P. intermedia* immediately post-treatment relative to baseline (*P* = 0.003). Inter-group analysis demonstrated significant differences for *P. intermedia* and *T. denticola* (*P* = 0.001 and 0.004), with both laser-assisted groups outperforming the SRP group. At 3 months, CAL and PPD demonstrated significant inter-group differences, while BOP did not.

**Conclusions:**

Laser-assisted periodontal therapy based on LANAP^®^-derived principles, using either an Nd:YAG 1064 nm or a 940 nm diode laser, offers superior immediate microbiological suppression and more favorable clinical outcomes than SRP alone.

**Trial registration:**

This trial was retrospectively registered at ClinicalTrials.gov; trial registration number: NCT06509412; date of registration: 19 July 2024.

**Supplementary Information:**

The online version contains supplementary material available at 10.1186/s12903-026-08676-x.

## Introduction

Periodontitis is a biofilm-mediated inflammatory condition affecting tooth-supporting structures, leading to irreversible periodontal tissue destruction if the underlying etiological factors are not eliminated [1]. It is acknowledged as a significant public health issue owing to its widespread global incidence and infectious-inflammatory characteristics [2].

The disease’s pathogenesis involves a molecular cascade that activates host-derived proteinases. These enzymes contribute to periodontal ligament fiber degradation and junctional epithelium apical migration, thereby facilitating apical extension of the dysbiotic subgingival biofilm along the root surface [1, 3]. Notably, this dysbiotic subgingival shift is considered a key driver of destructive host responses and periodontitis progression [4, 5].

The Expanded Human Oral Microbiome Database (eHOMD) [6] indicates that the oral microbiota encompasses roughly 774 bacterial species. Of these, around 58% are officially identified, 16% are cultivated yet not formally named, and approximately 26% are categorized as uncultivated phylotypes. Contemporary evidence has identified five periodontopathic species that disrupt the normal oral ecosystem and are typically targeted in chairside diagnostic assays: Porphyromonas gingivalis, Treponema denticola, Tannerella forsythia, Prevotella intermedia, and Aggregatibacter actinomycetemcomitans [7–9]. These species are frequently associated with periodontitis progression and irreversible periodontal tissue destruction [10–12].

Stage II periodontitis, as defined by the 2017 World Workshop classification [4], represents established moderate periodontitis with measurable attachment and bone loss without the complexity of advanced stages. This makes it a key window for effective nonsurgical and minimally invasive periodontal therapy and stabilization before tooth-threatening progression.

Accurate diagnosis of the severity and complexity, along with a risk assessment of periodontitis progression, is paramount for developing a tailored treatment strategy for each individual patient [13]. Real-time qPCR is widely acknowledged as a rapid and highly sensitive technique for the identification and quantification of different microbial species [14]. Most assays target the bacterial 16 S rRNA gene, a small-subunit sequence present in multiple copies in all bacterial genomes, containing conserved yet species-distinct regions that facilitate precise microbial differentiation [15].

Periodontal treatment primarily aims to control the inflammatory response by either surgical or non-surgical reduction of the burden of disease-associated microorganisms [13, 16, 17]. Most periodontitis patients respond favorably to non-surgical therapy based on supragingival plaque control and subgingival debridement [18]. However, despite being the cornerstone of periodontal therapy [13], SRP may be limited by constrained anatomy, where instrument access is restricted, and it often leaves a residual smear layer that harbors bacteria, endotoxins, and infected cementum [19]. Consequently, adjuvant strategies including locally or systemically delivered antibiotics and antiseptics [20], as well as non-pharmacologic modalities such as photodynamic therapy [21] and cold atmospheric plasma [22], have been suggested to improve biofilm suppression. Nevertheless, apprehensions regarding the adverse effects of previous approaches and inconsistent clinical benefits drive the search for alternative adjunctive approaches.

Currently, there is a growing emphasis on minimally invasive treatment strategies aimed at promoting periodontal regeneration [23]. The hallmark of periodontal regeneration is the reconstruction of a functional tooth-supporting attachment complex on a root surface previously affected by disease, histologically evidenced by new cementum deposition with inserted periodontal ligament fibers and new supporting alveolar bone [24].

Against this background, periodontal lasers have been investigated as adjuncts to SRP for their ability to selectively de-epithelialize the inflamed pocket lining, provide photo-antisepsis against pigmented anaerobes, and stabilize the wound environment via photothermal coagulation [25], with diode and Nd:YAG lasers being among the most frequently used systems in this context [26].

Laser-Assisted New Attachment Procedure (LANAP^®^) is a proprietary minimally invasive approach that is conventionally performed using a 1,064 nm Nd:YAG laser within a dedicated system. It incorporates selective de-epithelialization of the sulcular lining, meticulous root detoxification with ultrasonics, and stabilization of a fibrin clot to promote a stable wound environment [27]. Owing to its affinity for pigmented chromophores such as haemoglobin and melanin, 1,064 nm Nd:YAG may promote efficient coagulation, haemostasis, soft-tissue penetration, in addition to antimicrobial and anti-inflammatory benefits during periodontal therapy [27, 28]. Nonetheless, randomized controlled trials incorporating microbiological endpoints for LANAP^®^ remain limited, and the available evidence is heterogeneous [29–31].

Diode lasers are frequently used in the dental field due to their affordability, portability, and simplicity of operation [32]. Owing to their near-infrared wavelengths, they penetrate soft tissues and preferentially interact with pigmented chromophores, thereby supporting selective ablation of inflamed granulation tissue and pigmented periodontopathogens [27]. Sameera et al. [33] reported that a 940 nm diode-laser protocol analogous to LANAP^®^ was associated with improved periodontal healing reflected by superior clinical and radiographic outcomes, although early revascularization was slightly delayed. Nevertheless, diode-laser protocols remain heterogeneous, and direct microbiological comparisons with Nd:YAG-based approaches are scarce.

Therefore, the present study addressed the lack of direct comparative evidence on the immediate microbiological effects of 1064 nm Nd:YAG laser-assisted periodontal therapy performed according to LANAP^®^-derived principles, 940 nm diode laser-assisted minimally invasive protocol, and SRP alone in stage II periodontitis.

Accordingly, this three-arm, parallel-group, randomized controlled trial was designed to compare these three interventions. The primary objective was to compare their immediate effects on subgingival levels of *T. denticola* and *P. intermedia*, while the secondary objective was to compare their clinical outcomes, including CAL, PPD, and BOP, at 3-month follow-up. The null hypothesis (H₀) was that the three interventions would produce comparable reductions in *T. denticola* and *P. intermedia* and yield similar changes in CAL, PPD, and BOP.

## Materials and methods

### Study design

**Fig. 1:**
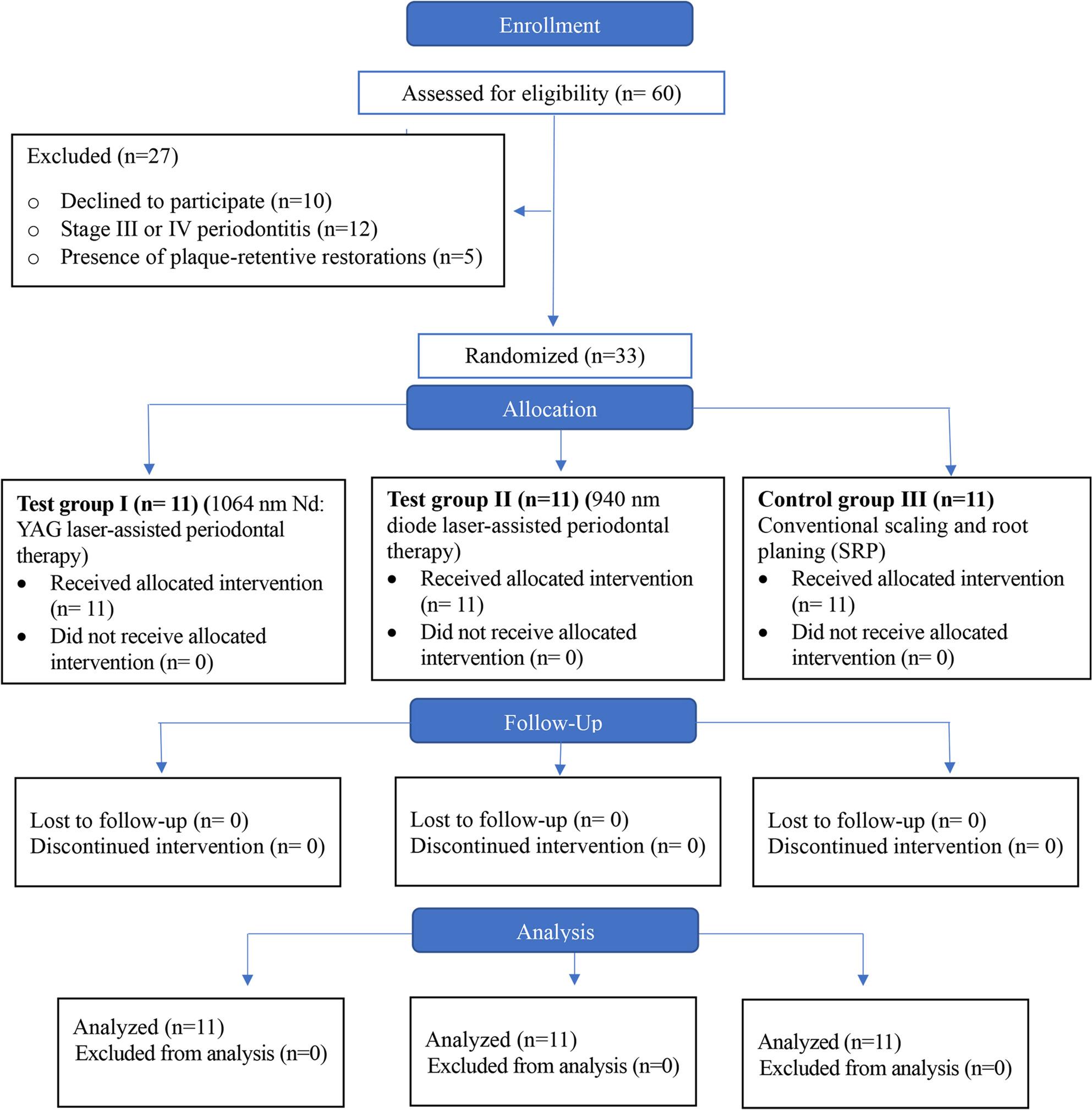
CONSORT® flow chart of the study

We executed a parallel-design, three-arm randomized controlled clinical trial that enrolled 33 eligible individuals with stage II periodontitis. Eligible participants were consecutively screened at the outpatient clinic of the Department of Oral Medicine, Periodontology, Diagnosis, and Oral Radiology, Faculty of Dentistry, Alexandria University, Egypt. The study was conducted from 1 January 2024 to 17 December 2025 (Fig. 1) [34].

### Ethical approval and informed consent

The Research Ethics Committee of the Faculty of Dentistry, Alexandria University, Egypt, authorized the research protocol (IRB No. 00010556; IORG No. 0008839). In keeping with international research ethics, the trial was implemented in line with the ethical standards of the Declaration of Helsinki (2013 revision) [35] and reported following the CONSORT 2010 guidelines for randomized controlled trials [34]. Prior to participation, all eligible participants were fully apprised of the study aims, nature, and possible risks and benefits, and provided written informed consent. Furthermore, this trial was registered at the U.S. National Institutes of Health Clinical Trials registry (ID: NCT06509412).

### Inclusion and exclusion criteria

Participants were recruited if they met the following criteria:

- Systemically healthy men and women aged between 30 and 60 years old.
- Adequate baseline oral hygiene, defined as an O’Leary plaque control record < 10% [36].
- Stage II periodontitis patients [4].
- Clinical attachment level of 3–4 mm and PPD ≥ 4 mm [4].
- Horizontal bone loss exceeding 15% confirmed radiographically [4].

### While exclusion criteria included

- Patients with furcation involvement or tooth mobility.
- Patients had received systemic antibiotic therapy within the preceding six months.
- Current smokers or alcohol users.
- Patients had undergone periodontal surgery within the previous 12 months.
- Pregnant or breastfeeding women.

### Sample size estimation

Sample size was calculated separately based on the reduction of bacterial counts (primary outcome) and CAL (secondary clinical outcome) to ensure adequate power for both outcomes. The final sample size was determined based on the larger of the two estimates to avoid underpowering of any outcome. The sample size was generated utilizing G*Power (Version 3.1.9.4), assuming a statistical power of 80% and a significance level of 5% based on previously reported mean reductions in clinical attachment loss of 1.24 (SRP), 2.02 (diode laser) [37], 0.44 (Nd:YAG laser) [28], and using the highest reported standard deviation (SD = 1) [28]. Based on an F-test requiring an effect size of 0.653, the sample size was estimated at 9 per group, increasing to 11 to account for potential attrition. The total required sample size was 33 participants [38].

### Grouping and randomization of the study participants

Thirty-three participants meeting the eligibility criteria for Stage II periodontitis were enrolled and allocated in a 1:1:1 ratio to three groups (*n* = 11). Group I (test I) underwent Nd:YAG 1064 nm laser-assisted periodontal therapy performed according to clinical principles described for the LANAP^®^ protocol [39, 40]; group II (test II) received 940 nm diode-laser assisted periodontal therapy using a minimally invasive protocol analogous to LANAP^®^ [33, 41], and group III (control) received conventional SRP using ultrasonic and hand instruments. To ensure unbiased random allocation, the random allocation sequence was generated by an independent statistician using a computer-generated random number sequence (Sealedenvelope™ simple randomiser tool). After eligibility confirmation and collection of baseline records, eligible participants were enrolled and assigned to the interventions by the principal operator using sequentially numbered, opaque, sealed envelopes (SNOSE), which were unsealed sequentially only at the time of intervention to reveal the group allocation. Due to the nature of the interventions, blinding of the participants and operators was not feasible. However, outcome assessors were blinded to group allocation. For statistical analysis, group assignments were coded as Groups I, II, and III, and the statistician remained blinded to the actual treatment identities until the analysis was completed.

### Intervention

Following the collection of baseline records for both microbiological samples and clinical parameters, patients assigned to the laser-assisted groups (Nd:YAG-based or diode-based minimally invasive protocols) underwent the procedure under local anaesthesia to ensure comfort and reduce thermal sensitivity during laser application. Furthermore, laser wavelength-specific protective eyewear was worn by the patient, operator, and dental assistant throughout the procedure to prevent ocular exposure to laser radiation. All study interventions were subsequently performed by the same trained operator using predefined laser settings and a standardized treatment protocol, thereby minimizing inter-operator variability.

**Fig. 2:**
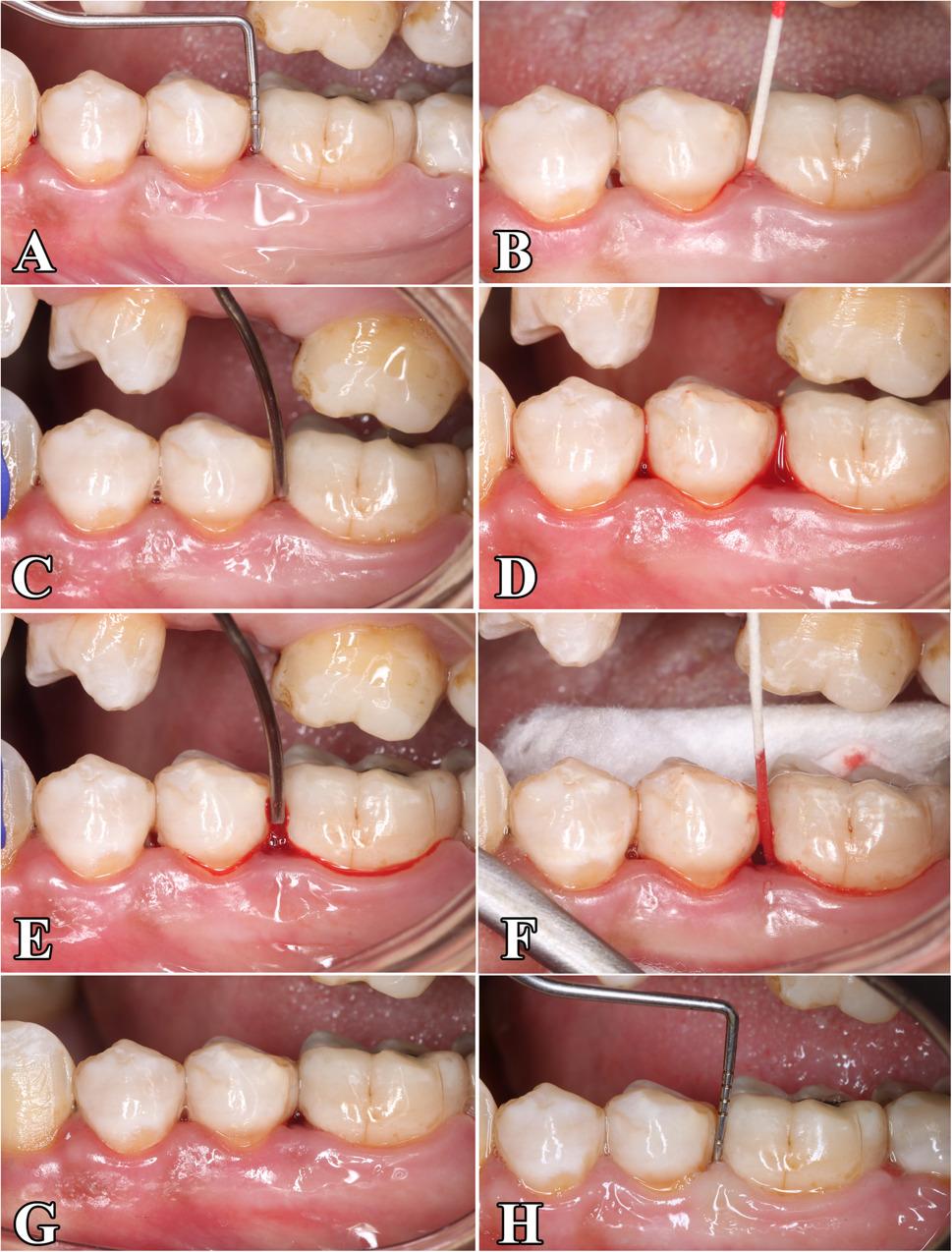
1064 nm Nd:YAG laser-assisted periodontal therapy. **A** Baseline PPD. **B** Baseline 1st sub-gingival biofilm microbial sample. **C** 1st pass of 1064 nm Nd:YAG laser (troughing). **D** Fresh blood after SRP. **E** 2nd pass of 1064 nm Nd:YAG laser. **F** Immediate post-operative 2nd sub-gingival microbial sample. **G** Blood clot formation. **H** Three-month follow-up PPD

Test group I underwent Nd:YAG 1064 nm laser-assisted periodontal therapy following the clinical sequence described for LANAP^®^ [40], without using the proprietary LANAP^®^ system. The Nd:YAG laser (Fotona d.o.o., Ljubljana, Slovenia) was used. The protocol consisted of two standardized laser passes, as shown in Supplementary Material 1: Supplementary Table 1. The first pass, known as laser troughing, the laser was set to 3 W at 20 Hz in micro short-pulse mode (100 µs pulse duration) [39]. A 320 μm fiber was advanced circumferentially along the pocket in a coronal-apical direction with horizontal sweeping strokes parallel to the root surface, 1 mm shorter than the probing depth, to selectively ablate the pocket epithelium and disrupt inflamed soft tissue [40]. Thereafter, an ultrasonic scaler was used to remove sub-gingival calculus and infected cementum from the root surfaces. Subsequently, the second laser pass was performed with the following parameters: 4 W power, very-long-pulse mode (600 µs pulse duration), and 20 Hz frequency [39]. This pass was applied in an apical-coronal direction to enhance the fibrin clot formation and stabilization, promoting healing from the inside out [40, 42, 43]. Finally, this fibrin clot was compressed against the root surface [40] (Fig. 2).

**Fig. 3:**
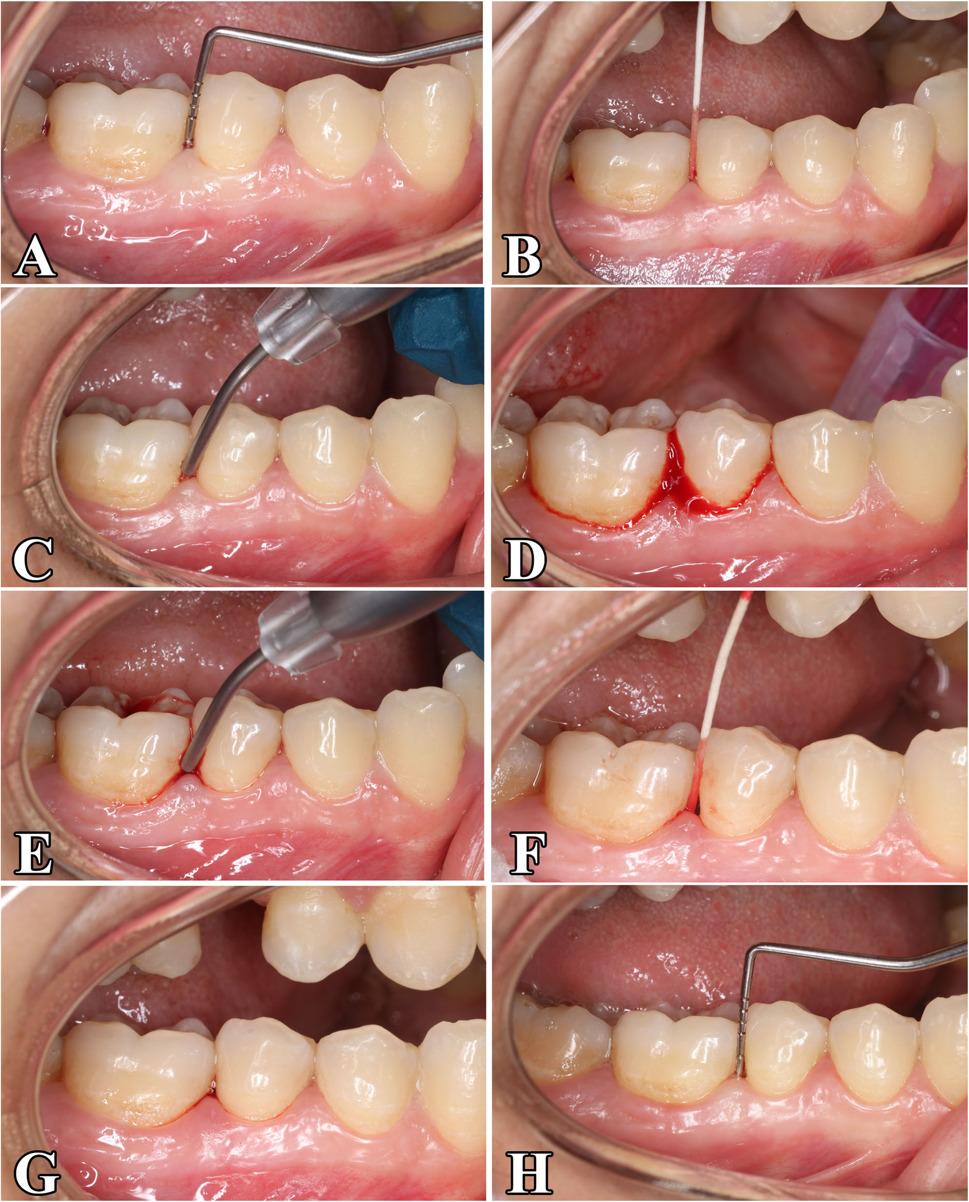
940 nm diode laser-assisted periodontal therapy. **A** Baseline PPD. **B** Baseline 1st sub-gingival biofilm microbial sample. **C** 1st pass of 940 nm diode laser (troughing). **D** Fresh blood after SRP. **E** 2nd pass of 940 nm diode laser. **F** Immediate post-operative 2nd sub-gingival microbial sample. **G** Blood clot formation. **H** Three-month follow-up PPD

Test group II received periodontal therapy using a diode laser operating at 940 nm (BIOLASE Inc., Foothill Ranch, CA, USA), following a protocol modeled on the clinical steps of LANAP^®^ but adapted to diode-laser parameters [33]. The protocol consisted of two laser passes, as detailed in Supplementary Material 1: Supplementary Table 1. The first laser pass was delivered through a 300 μm flexible fiber-optic cable at a power setting of 1 W in continuous mode to de-epithelialize the infected pocket lining [41], followed by ultrasonic scaling to remove subgingival calculus and infected root cementum. After that, the second laser pass was performed at 0.5 W in continuous mode to promote fibrin clot formation and disinfect the site [33]. Finally, this fibrin clot was compressed against the root surface [40] (Fig. 3).

**Fig. 4:**
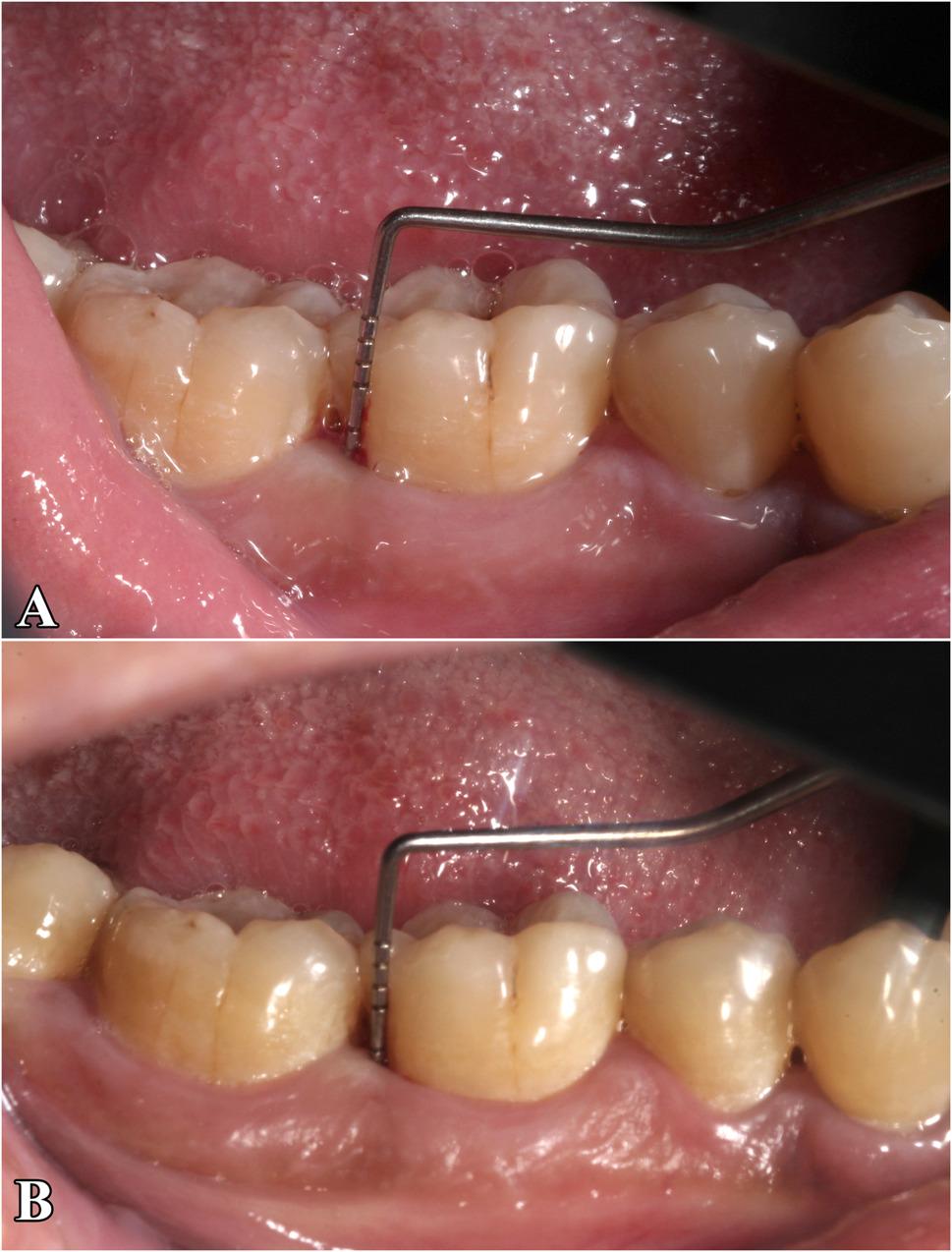
Scaling and root planing. **A** Baseline PPD, (**B**) Three-month follow-up PPD

Patients assigned to control group III experienced SRP utilizing an ultrasonic device (Woodpecker USD-P LED Ultrasonic Scaler, Ende, Information Industrial Park, National High-Tech Zone, Guilin, Guangxi, 541004, P.R. China) at a moderate power setting with appropriate scaling tips. Additionally, universal manual curettes (HuFriedy^®^ Group, Chicago, IL, USA) were employed to achieve smooth root surfaces, with complete removal of visible and tactile calculus or diseased cementum [44] (Fig. 4).

Immediately following each treatment approach, the second subgingival microbial samples were collected [40]. All patients were instructed to use 0.12% chlorhexidine mouthwash twice daily for 2 weeks, following 1 h of tooth -brushing using the modified Bass technique with a new soft-bristled toothbrush and fluoride toothpaste. Follow-up for oral hygiene maintenance was done at 2 weeks, 1 month, and 3 months.

### Clinical parameter assessment

Periodontal clinical measurements of PPD, CAL, and BOP were evaluated at baseline and at three months following treatment procedures using the same calibrated William’s probe (KLS Martin SE & Co. KG, Tuttlingen, Germany) to standardize measurements by blinded, pre-calibrated clinicians [45, 46]. Prior to study initiation, two clinicians underwent calibration, and both intra- and inter-examiner agreement were verified; kappa coefficients ranged from 0.82 to 0.88, reflecting excellent reproducibility of measurements across time [47].

### Microbiological processing

Subgingival biofilm samples were obtained from the site exhibiting the deepest probing depth using a standardized sampling approach [48]. After isolating the sampling area with cotton rolls and mechanically removing supragingival plaque, sterile paper points were introduced into each pocket for 10 s at both baseline and immediately after each intervention [40] to analyse two bacterial species: *T. denticola and P. intermedia*. For laser-treated sites, paper points were placed and removed before complete fibrin-clot formation following the second laser pass, and before soft-tissue compression to avoid disruption of early wound stabilization [40]. Next, paper points were put in microcentrifuge tubes containing 1 mL sterile saline and transported to Alexandria Main University Hospital Microbiology laboratory, where tubes were preserved at -20 °C until DNA extraction, after which bacterial quantification was performed using real-time (qPCR) following the methodology described by Tomova et al. [49].

Prior to DNA extraction, each microcentrifuge tube was vortex-mixed for approximately 5 min to ensure adequate sample dispersion. A 200 µL volume of the resulting suspension was then processed for genomic DNA isolation using the QIAamp DNA Mini Kit (Qiagen, Germany). Commercially available primers (Invitrogen; Thermo Fisher Scientific) were used for amplification (Table 1). For each sample, 8 µL of extracted DNA was added to a 20-µL reaction mixture containing 10 µL of 2× Maxima SYBR Green master mix and 10 pmol of forward primer and 10 pmol of reverse primer, Amplification was performed on the Rotor-Gene Q (QIAGEN, Germany) after an initial denaturation step at 95 °C for 10 min, succeeded by 40 cycles of 95 °C for 30 s, annealing at 57 °C for 30 s, and ended by extension at 72 °C for 30 s. Product specificity was assessed by melt-curve analysis from 40 °C to 95 °C using 1 °C increments with a 10-s hold at each step. Relative quantification of targeted bacterial DNA was automatically determined by the Rotor-Gene software using cycle threshold (Ct) values for each target bacterium normalized to the Ct of universal bacterial DNA. Results were expressed as relative fold differences rather than absolute copy numbers.

**Table 1:** Oligonucleotide primer sequences of the bacteria

Bacteria	Primer Sequence (5′–3′)
(16 S rRNA) [50]	Forward: CGCTAGTAATCGTGGATCAGAATG
	Reverse: TGTGACGGGCGGTGTGTA
*Prevotella intermedia* [51]	Forward: GACCCGAACGCAAAATACAT
	Reverse: AGGGCGAAAAGAACGTTAGG
*Treponema denticola* [52]	Forward: CGGGCGTGCATCTTGTCGTCTAC
	Reverse: CTTAACCGGCCGCCTCTTTGAA

To exclude cross-contamination and carryover, both negative extraction controls (sterile distilled water substituted for the sample during extraction) and no-template controls (sterile distilled water substituted for DNA extract in the reaction mix) were included in each run. The analytical sensitivity of the real-time qPCR assay, including DNA extraction efficiency, was evaluated using sterile saline spiked with tenfold serial dilutions of *Streptococcus mutans* (ATCC 25175), down to 1 colony-forming unit/mL.

### Statistical analysis

Statistical analyses were performed with SPSS version 23 for Windows (Armonk, NY, USA). Distributional assumptions were evaluated using the Shapiro–Wilk test and Q–Q plots. A normal distribution was confirmed only for age, which was analyzed using one-way ANOVA, whereas bacterial load showed non-normal distributions even after log transformation. Quantitative variables were expressed as mean ± standard deviation (SD) and median with interquartile range (IQR) as appropriate. Categorical variables (gender and bleeding) were presented as counts and percentages and analyzed using the Pearson Chi-Square test. The McNemar test was employed to assess changes in bleeding status post-treatment. The Kruskal-Wallis test was used to compare all outcomes across the three groups, and the Wilcoxon signed-rank test was applied for within-group pre- and post-treatment comparisons. Dunn’s post hoc test was used for pairwise comparisons between groups with Bonferroni correction to adjust for type I error. Percent change was calculated according to the formula [(post-treatment values – pretreatment values)/pretreatment values] x 100]. A p-value < 0.05 was considered statistically significant.

## Results

Thirty-three patients with stage II periodontitis completed the study and were allocated equally to the three treatment groups (*n* = 11 per group). Baseline demographic characteristics were comparable across the three groups. No significant differences were observed in age (*p* = 0.458) or sex distribution (*p* = 0.886), indicating successful baseline randomization (Table 2).

**Table 2:** Demographic data of the study groups

		Group I (*n* = 11)	Group II (*n* = 11)	Group III (*n* = 11)	Test (*p*-value)
Age in years	Mean ± SD	43.7.42 ± 7.43	40.64 ± 4.74	40.91 ± 4.68	0.802^#^ (0.458)
Gender: n (%)	Males	5 (45.5%)	6 (54.5%)	5 (45.5%)	0.243^§^ (0.886)
	Females	6 (54.5%)	5 (45.5%)	6 (54.5%)	

#One Way ANOVA test, §Pearson Chi Square test

### *Prevotella intermedia*

**Fig. 5:**
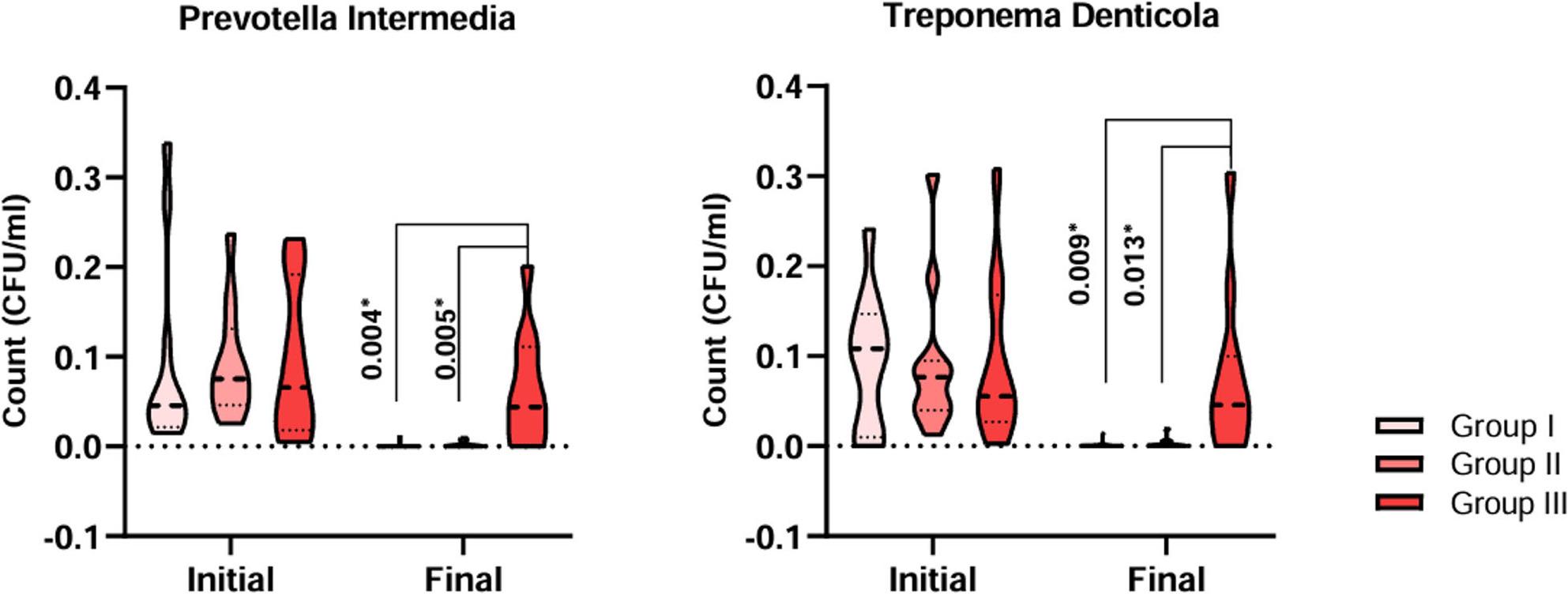
Baseline and post-treatment bacterial counts for P. intermedia and T. denticola among groups

Regarding *P. intermedia*, the log relative concentration did not differ significantly among the three groups at baseline (*p* = 0.591). Immediately following intervention, all groups exhibited a significant within-group reduction (*p* = 0.003 for each group) and inter-group difference (*p* = 0.001). Specifically, the Nd:YAG- and diode-based minimally invasive laser protocols (Groups I and II) achieved markedly lower post-treatment median values than SRP alone (Group III). Median values reached 0.00 in Groups I and II, whereas Group III (SRP) exhibited 0.02. Percent-reduction analysis confirmed this pattern, with a median percent reduction of 100.00% in laser-assisted groups on the relative qPCR scale used for analysis, compared with 35.87% in the SRP group (Table 3). Pairwise comparisons further indicated significantly lower post-treatment *P. intermedia* levels in Groups I and II vs. Group III (*p* = 0.004 and 0.005, respectively), while no difference was detected between the two laser protocols (*p* = 1.00) (Fig. 5). The same pattern was observed for percent reduction, favoring laser groups I and II over SRP (*p* = 0.004 and 0.005, respectively) (Table 5).

**Table 3:** Comparison of log relative concentration of bacterial load among the study groups

		Group I (*n* = 11)	Group II (*n* = 11)	Group III (*n* = 11)	Test^1^(*p*-value)
Prevotella intermedia					
Pre	Mean Rank	15.09	19.27	16.64	1.052 (0.591)
	Median (Min - Max)	0.02 (0.01–0.13)	0.03 (0.01–0.09)	0.03 (0.00–0.09)	
	Mean ± SD	0.04 ± 0.04	0.04 ± 0.02	0.04 ± 0.03	
Post	Mean Rank	12.86	13.18	24.95	13.333 (0.001*)
	Median (Min - Max)	0.00^a^ (0.00–0.01)	0.00^a^ (0.00–0.00)	0.02^b^ (0.00–0.08)	
	Mean ± SD	0.00 ± 0.00	0.00 ± 0.00	0.03 ± 0.02	
**Test** ^**2**^ **(** ***p*** **-value)**		2.934 (0.003*)	2.934 (0.003*)	2.934 (0.003*)	
Percent reduction	Mean Rank	12.91	13.09	25.00	13.479 (0.001*)
	Median (Min - Max)	100.00 (41.23–100)^a^	100.00 (70.31 − 100.00)^a^	35.87 (8.33–100.00)^b^	
	Mean ± SD	94.51 ± 17.68	96.07 ± 9.17	47.50 ± 34.67	
*Treponema denticola*					
Pre	Mean Rank	17.55	16.86	16.59	0.057 (0.972)
	Median (Min - Max)	0.04 (0.00–0.09)	0.03 (0.01–0.11)	0.02 (0.00–0.12)	
	Mean ± SD	0.04 ± 0.03	0.04 ± 0.03	0.04 ± 0.03	
Post	Mean Rank	13.18	13.64	24.18	11.259 (0.004*)
	Median (Min - Max)	0.00^a^ (0.00–0.01)	0.00^a^ (0.00–0.01)	0.02^b^ (0.00–0.12)	
	Mean ± SD	0.00 ± 0.00	0.00 ± 0.00	0.03 ± 0.03	
**Test** ^**2**^ **(** ***p*** **-value)**		2.934 (0.003*)	2.934 (0.003*)	2.934 (0.003*)	
Percent reduction	Mean Rank	13.09	13.73	24.18	11.274 (0.004*)
	Median (Min - Max)	100.00 (91.41–100)^a^	100.00 (59.29–100)^a^	10.67 (1.30–100.00)^b^	
	Mean ± SD	98.98 ± 2.57	93.76 ± 13.33	37.93 ± 41.94	

* Statistically significant difference at *p* value < 0.05, Test^1^: Kruskal-Wallis test, Test ^2^: Wilcoxon signed-rank test, different superscript lowercase letters denote statistically significant differences between groups

### *Treponema denticola*

Regarding *T. denticola*, the baseline log relative concentration was comparable across the three groups (*p* = 0.972). Immediately following treatment, all groups showed a significant within-group reduction (*p* = 0.003 for each group). However, post-treatment *T. denticola* levels differed significantly between groups (*p* = 0.004), with both laser-assisted protocols (Groups I and II) demonstrating superior suppression than SRP alone (Group III). The post-treatment median was 0.00 in Groups I and II, whereas Group III remained residually detectable, with a post-treatment median of 0.02. Percent-reduction analysis further reinforced this separation, with a median percent reduction of 100.00% in Groups I and II on the relative qPCR scale used for analysis, and 10.67% in Group III (Table 3). Pairwise comparisons demonstrated significantly lower post-treatment T. denticola levels in Groups I and II relative to Group III (*p* = 0.009 and 0.013, respectively), with no difference between the two laser protocols (*p* = 1.00) (Fig. 5). Likewise, percent reduction was significantly superior in the laser groups than SRP (Groups I and II versus Group III, *p* = 0.008 and 0.014, respectively), while remaining comparable between the two laser protocols (*p* = 1.00) (Table 5).

### Clinical attachment level (CAL)

With respect to clinical attachment level (CAL), baseline values were comparable across the three groups (*p* = 0.790). At the 3-month evaluation, CAL differed significantly between groups (*p* < 0.001), with lower post-treatment CAL in Group I (median 1.0 mm) and Group II (median 2.0 mm) than in Group III (median 3.0 mm). Within-group analyses showed significant CAL improvements in laser-assisted groups, Group I (*p* = 0.002) and Group II (*p* = 0.003), whereas the reduction in Group III was not statistically significant (*p* = 0.083) (Table 4). Consistently, the percent reduction in CAL revealed a significant between-group difference (*p* < 0.001), with greater median reductions in Group I (66.67%) and Group II (50.00%) than in Group III (0.00%) (Table 4). Pairwise analysis indicated a significantly better post-treatment CAL in both Group I and Group II than Group III (Group I vs. III: *p* < 0.001 for both post-CAL and change; Group II vs. III: *p* = 0.002 for post-CAL and change), with no difference detected between the laser-assisted groups (Table 5).

**Table 4:** Comparison of clinical attachment level (CAL), probing pocket depth (PPD), and bleeding on probing (BOP) among the study groups

		Group I (*n* = 11)	Group II (*n* = 11)	Group III (*n* = 11)	Test^1^(*p*-value)
CAL					
Pre	Mean Rank	16.00	17.50	17.50	0.471 (0.790)
	Median (Min - Max)	3.00 (3.00–4.00)	3.00 (3.00–4.00)	3.00 (3.00–4.00)	
	Mean ± SD	3.09 ± 0.30	3.18 ± 0.41	3.18 ± 0.41	
Post	Mean Rank	10.14	14.14	26.73	20.948 (< 0.001*)
	Median (Min - Max)	1.00^a^ (1.00–2.00)	2.00^a^ (1.00–3.00)	3.00^b^ (2.00–4.00)	
	Mean ± SD	1.27 ± 0.47	1.64 ± 0.67	2.91 ± 0.54	
**Test** ^**2**^ **(** ***p*** **-value)**		3.127 (0.002*)	3.017 (0.003*)	1.732 (0.083)	
**Percent reduction**	Mean Rank	9.95	13.82	27.23	21.23 (< 0.001*)
	Median (Min - Max)	66.67^a^ (33.33–66.67)	50.00^a^ (25.00–66.67)	0.00^b^ (0.00–33.33)	
	Mean ± SD	59.09 ± 13.67	49.24 ± 17.66	8.33 ± 14.43	
PPD					
Pre	Mean Rank	15.64	17.14	18.23	1.222 (0.543)
	Median (Min - Max)	5.00 (4.00–6.00)	5.00 (4.00–6.00)	5.00 (5.00–6.00)	
	Mean ± SD	5.00 ± 0.45	5.09 ± 0.70	5.18 ± 0.41	
Post	Mean Rank	11.36	15.95	23.68	11.733 (0.003*)
	Median (Min - Max)	3.00^a^ (2.00–4.00)	3.00^ab^ (2.00–5.00)	4.00^b^ (3.00–5.00)	
	Mean ± SD	3.00 ± 0.45	3.36 ± 0.92	4.09 ± 0.70	
**Test** ^**2**^ **(** ***p*** **-value)**		3.317 (0.001*)	3.071 (0.002*)	2.585 (0.010*)	
**Percent reduction**	Mean Rank	11.09	15.73	24.18	11.57 (0.003*)
	Median (Min - Max)	40.00^a^ (33.33–50.00)	40.00^ab^ (16.67–50.00)	20.00^b^ (0.00–40.00)	
	Mean ± SD	40.30 ± 3.79	34.85 ± 11.58	20.61 ± 15.33	
BOP					
Pre	Yes	11 (100%)	11 (100%)	11 (100%)	NA
	No	0 (0%)	0 (0%)	0 (0%)	
Post	Yes	2 (18.2%)	3 (27.3%)	4 (54.5%)	0.917 (0.632)
	No	9 (81.8%)	8 (72.7%)	7 (45.5%)	
**Test** ^**2**^ **(** ***p*** **-value)**		(0.004*)	(0.008*)	(0.016*)	

* Statistically significant difference at *p-value* < 0.05. For CAL and PPD, Test 1: Kruskal-Wallis test; Test 2: Wilcoxon signed-rank test. Different lowercase superscript letters denote statistically significant differences between groups. For bleeding on probing, Test^1^: the Pearson Chi-Square test, Test ^2^: the McNemar test

**Table 5:** Pairwise comparison of clinical attachment level (CAL), probing pocket depth (PPD), and bacteria between the study groups

Groups	Compared to	*p*-value					
		CAL (post)	PPD (post)	Change in CAL	Change in PPD	Prevotella intermedia (post)	Treponema denticola (post)
						Percent reduction	Percent reduction
Group I	Group II	0.580	0.167	0.978	0.704	1.00	1.00
	Group III	< 0.001*	0.002*	< 0.001*	0.002*	0.004*	0.008*
Group II	Group III	0.002*	0.807	0.002*	0.091	0.005*	0.014*

*Statistically significant difference at p-value < 0.05. Dunn’s post hoc test

### Probing pocket depth (PPD)

At baseline, PPD was comparable among the three groups (*p* = 0.543). After 3 months, a significant between-group difference was identified (*p* = 0.003), with Group I and II showing a lower median PPD (3.0 mm) than Group III (4.0 mm). Within-group comparisons demonstrated significant reductions in PPD across all groups (*p* = 0.001, 0.002, 0.010, respectively) (Table 4). Consistently, the percent reduction in PPD differed significantly across groups (*p* = 0.003), with greater median reductions in Groups I and II (40.0%) compared with Group III (20.0%) (Table 4). Pairwise comparisons showed that only Group I achieved significantly better post-treatment PPD and a significantly greater percent reduction in PPD than Group III (*p* = 0.002 for post-PPD and change) (Table 5).

### Bleeding on probing (BOP)

Bleeding on probing (BOP) was positive in all groups at baseline (100%). At 3-month follow-up, bleeding decreased to 18.2% in Group I, 27.3% in Group II, and 54.5% in Group III, with no significant between-group difference (*p* = 0.632). However, within-group analyses showed significant reductions from baseline in all groups (*p* = 0.004, 0.008, and 0.016, respectively) (Table 4).

Accordingly, the immediate microbiological reductions observed in the laser-treated groups were accompanied by more favorable short-term clinical outcomes, particularly with respect to CAL. Both laser-assisted groups showed lower post-treatment CAL values than the SRP at three-month follow-up.

## Discussion

The present three-arm randomized controlled trial demonstrated that both 1064 nm Nd:YAG and 940 nm diode laser-assisted minimally invasive periodontal therapy, following LANAP^®^ principles, were associated with greater immediate suppression of the relative qPCR-derived levels of *T. denticola* and *P. intermedia* compared with SRP alone. Although all groups showed significant within-group improvement, the laser-treated groups demonstrated significantly lower post-treatment relative microbial values and superior short-term clinical attachment gain at 3 months than SRP alone. These findings suggest that adjunctive near-infrared laser therapy may provide incremental microbiological and clinical benefits in the treatment of stage II periodontitis.

Laser-assisted minimally invasive periodontal therapy has been proposed to enhance decontamination and early wound stabilization through selective interaction with pigmented inflamed tissues and photothermal antimicrobial effects [53]. These biological properties, when combined with mechanical debridement, may partly explain the pronounced immediate suppression of *T. denticola* and *P. intermedia*, as well as the superior short-term clinical attachment gain observed in the laser-assisted groups.

Building on that biological foundation, Giannelli et al. [54] noted that periodontopathogens may remain intracellularly within tissues near the pocket epithelium after conventional therapy. They further suggested that Nd:YAG and diode systems may eliminate periodontopathogenic bacteria residing inside the gingival epithelial cells.

Consequently, the efficacy of Nd:YAG and diode-based laser-assisted minimally invasive periodontal therapy following LANAP^®^ principles, compared with conventional SRP on subgingival levels of *P. intermedia* and *T. denticola*, alongside CAL, PPD, and BOP in stage II periodontitis, constituted the rationale for the present investigation.

In the current trial, subgingival *T. denticola and P. intermedia* showed a clear distinction between laser-assisted periodontal therapy and conventional SRP. Despite significant immediate post-treatment reductions across all groups (*p* = 0.003), the laser-treated groups showed higher percent-reduction medians on the relative qPCR scale used for analysis, reached 100% for both species, compared with 35.87% for *P. intermedia* and 10.67% for *T. denticola* in the SRP group. However, this marked suppression should not be interpreted as absolute eradication of the targeted pathogens.

These findings align with the wider microbiological literature, which reported that mechanical debridement can diminish anaerobic periodontal pathogens but may not reliably achieve complete short-term eradication when assessed immediately after instrumentation [55–57].

Our Nd:YAG-based laser-assisted protocol results are consistent with McCawley et al. [40] who found that most LANAP^®^-treated patients became negative for red/orange-complex species immediately after therapy (17/20; 85%), whereas this occurred infrequently following ultrasonic debridement alone (1/6; 16.7%). In contrast, a distinct pilot study by McCawley et al. [31] reported that Nd:YAG laser monotherapy targeting subgingival biofilms produced an immediate reduction in red/orange-complex burden of approximately 59%, yet only a minority achieved culture negativity for all assessed species, including *P. intermedia* (25%). Notably, this effect was observed before SRP and the subsequent LANAP^®^ steps. Taken together, these two investigations by McCawley et al. [31, 40] suggest that the immediate microbiological effect of Nd:YAG may be more pronounced when integrated into a combined treatment sequence that includes mechanical debridement, rather than applied as monotherapy.

On the other hand, Bechir et al. [29] documented an overall improvement in clinical periodontal status after LANAP^®^, with a significant reduction in *P. gingivalis*, whereas no significant change was detected for *T. denticola*,* A. actinomycetemcomitans*, or total bacterial count (TBC) at the 6-week evaluation. However, the pronounced microbiological effect observed in our Nd:YAG-based group aligns with that reported in a comparative study by Caccianiga et al. [30], which confirmed limited bacterial responsiveness to conventional therapy and greater susceptibility to OHLLT and LANAP^®^.

In line with our diode-based minimally invasive protocol findings, Odor et al. [58] reported that SRP alone did not significantly reduce TBC at 1-month follow-up (*p* = 0.124), whereas adjunctive 940-nm diode irradiation with or without H₂O₂ photoactivation produced significant reductions (*p* = 0.001 and *p* < 0.001, respectively). However, at the species level, the pattern was not uniform: while *T. denticola* counts were comparable between the SRP group and the SRP + diode 940 nm group, *P. intermedia* counts showed a significant between-group difference. Our study, by comparison, suggests greater immediate relative reduction for both species with the sequential diode-based minimally invasive approach, which includes two laser applications separated by conventional SRP.

Agarwal et al. [59] Similarly, concluded that incorporating a 940-nm diode laser into modified Widman flap surgery resulted in significantly greater suppression of *P. intermedia*,* P. gingivalis*,* and A. actinomycetemcomitans* at 6-week and 6-month follow-up intervals relative to the sham laser. Similarly, El Mobadder et al. [56] found that combining 980-nm diode irradiation and 0.5% NaOCl irrigation with SRP achieved significantly greater immediate suppression of TBC, with complete eradication of several taxa, including *P. intermedia*, and lower residual levels of key anaerobes such *as T. denticola*, compared with SRP alone, which reduced but did not completely eliminate these organisms. However, this microbiological effect was associated with greater gingival recession in the diode adjunctive group.

Despite these positive outcomes, Pawelczyk-Madalińska et al. [60] and Patel et al. [61]highlighted in their systematic reviews of human RCTs and cohort studies that the microbiological effects of adjunctive diode lasers remain inconsistent, largely due to heterogeneity in treatment protocols and restricted assessment of microbiological outcomes, with several randomized trials [62–64] reporting no added pathogen reduction beyond SRP alone.

Furthermore, in the current three-arm trial, the clinical results across PPD, CAL, and BOP support two parallel realities of modern periodontal care: SRP is consistently effective, yet laser-assisted protocols can yield additive benefits. Regarding PPD and BOP, all groups demonstrated significant intra-group improvement by 3 months. This finding aligns with guideline-driven stepwise therapy, in which risk-factor control, oral hygiene reinforcement, and supra- and subgingival instrumentation form the basis for treating stage I–III periodontitis [13]. However, the key informative finding was that the laser-assisted protocols yielded outcomes beyond those associated with SRP alone. In the inter-group comparison, PPD differed significantly among groups (*p* = 0.003) and was significantly lower in the Nd:YAG-based laser-assisted protocol (Group I) than SRP (Group III) (*p* = 0.002), whereas the 940 nm diode-based laser-assisted protocol (Group II) did not significantly outperform SRP for PPD (*p* = 0.807).

With respect to CAL, both laser-assisted groups showed significant intra-group CAL gain in Group I and II (*p* = 0.002, 0.003, respectively), whereas SRP did not achieve a statistically significant attachment gain at 3 months (*p* = 0.083). Inter-group analysis further revealed that both laser groups achieved significantly greater CAL gain than SRP (*p* = 0.001 and 0.002, respectively), with no significant difference between the two laser-assisted groups (*p* = 0.580).

Importantly, although BOP decreased significantly within all groups, the inter-group difference at 3 months was not statistically significant. Therefore, the present findings do not support a clear adjunctive advantage of either laser protocol over SRP with respect to BOP.

Our Nd:YAG-based laser-assisted protocol outcomes are consistent with several controlled human studies reporting greater probing depth reduction and/or clinical attachment gains with the LANAP^®^ protocol compared with conventional instrumentation [42, 65, 66], although not all the studies have shown uniform clinical benefits [39, 67]. Yukna et al. [42] observed larger PPD reductions and greater CAL gains at 3 months in LANAP^®^-treated sites than in control sites, supporting the possible added clinical benefit of laser-assisted protocol beyond mechanical therapy alone. Similarly, in deep pockets (≥ 7 mm), Dadas et al. [66] reported that the LANAP^®^ and LLLT groups exhibited continued greater PPD reduction at 3 months than at 1 month, whereas SRP alone showed no further improvement beyond the 1-month outcome, alongside significantly greater CAL gains in the test groups (*p* < 0.001). Additionally, all groups demonstrated a significant reduction in BOP at the 3-month follow-up, with no significant intergroup differences at any point.

Nevertheless, Nevins et al. [65] reported sustained clinical improvement over 9 months, with a mean PPD decreasing from approximately 4.62 to 3.14 mm, a concomitant CAL gain of approximately 0.92 mm, and minimal recession of about 0.66 mm. In addition, Bechir et al. [68] reported that both LANAP^®^ and SRP approaches improved PPD, CAL, BOP, and plaque control at 6 weeks; however, LANAP^®^ demonstrated greater stability in sustaining these improvements over a 12-month period, whereas the SRP group indicated a slight decline in several outcomes, most notably in attachment levels.

Additional long-term clinical evidence of LANAP^®^ was introduced by Yukna et al. [69], who observed a significant reduction in PPD, improved CAL, and furcation status, with approximately 40% clinical closure of grade II furcation involvement and minimal recession of 0.1 mm at a 12–18 months follow-up period. Consequently, the authors suggested that LANAP^®^ may represent an effective, less invasive approach for managing periodontitis.

In contrast, Dortaj et al. [39] reported that although Nd:YAG-assisted therapy reduced PPD in residual pockets, it was associated with greater gingival recession and did not yield superior CAL gain versus control, suggesting that the additional PPD reduction was predominantly recession-driven rather than reflecting true attachment gain or pocket resolution. Similarly, Sgolastra et al. [67], in a meta-analysis, reported that adjunctive Nd:YAG significantly improved reductions in PD and GCF but did not confer a significant advantage in CAL gain or PI change.

Regarding the diode-based minimally invasive protocol clinical findings, several studies have investigated the diode lasers as an adjunctive modality to SRP in the management of chronic periodontitis [70–72]. In a single-masked split-mouth RCT, Seyed-Monir et al. [72] reported that although the adjunctive use of diode 940 nm showed a non-significant intergroup difference in PPD, tooth-level analysis demonstrated significantly greater CAL gains at first and second premolars and greater BOP reduction at second premolars versus SRP (*p* < 0.05).

Consistently, Sameera et al. [33] reported that despite delayed initial revascularization, the 940-nm diode-based LANAP exhibited significantly greater improvements in key clinical parameters PI, GI, PD, and CAL, along with radiographic bone level, compared with ENAP across follow-up visits. The authors attributed these superior outcomes to the proposed regenerative mechanisms of LANAP^®^, including selective photo-thermolysis of the inflamed pocket epithelium, formation of a stabilizing sticky fibrin clot at the pocket entrance, and bio-stimulatory effects that may promote recruitment and activation of progenitor cells, thereby facilitating healing in a coronal direction [33, 73, 74]. Moreover, Chandra et al. [37] reported that adding diode-LANAP to SRP produced significantly greater improvements in PPD, CAL, and GI at 90 days and a deeper microbiological reduction of *A. actinomycetemcomitans* and *P. gingivalis* in relation to SRP alone. However, the available evidence is not entirely consistent, Tabari et al. [41] found that adjunctive use of 940-nm diode laser therapy with SRP produced significant short-term (1-month) improvements in PPD, CAL, and BI compared with SRP alone; however, at 3 months, the adjunct benefit persisted only for BI, with no significant intergroup differences in PPD or CAL.

No treatment-related adverse effects were observed during the study period. Nevertheless, the findings of this trial should be interpreted in light of several limitations. The relatively small sample size, short clinical follow-up period, and restricted immediate post-operative microbiological assessment limited the evaluation of the durability of the observed effects and the potential for microbial recolonization. Accordingly, the external validity of the findings should be interpreted with caution. Future laser-assisted trials should therefore include larger populations and longer clinical and microbiological follow-up (≥ 6–12 months). Additionally, a broader panel of periodontal pathogens, particularly *Porphyromonas gingivalis*, should be assessed to further characterize the microbiological impact of the proposed laser-assisted parameters. Moreover, assessing inflammatory mediators in gingival crevicular fluid would help clarify potential host-modulatory effects.

## Conclusion

Within the study limitations, both 1064 nm Nd:YAG and 940 nm diode laser-assisted minimally invasive periodontal therapy following LANAP^®^ principles were associated with a greater immediate reduction in *P. intermedia* and *T. denticola* and short-term clinical improvement, particularly for CAL, compared with SRP alone. However, Further studies are needed to further define their longer-term microbiological and clinical significance. 

## Supplementary Information

Supplementary Material 1: Table 1. 1064 nm Nd:YAG and 940 nm diode laser parameters.

Supplementary Material 2: CONSORT checklist.

## Data Availability

All data included in this study are available from the corresponding author upon request.

## Abbreviations

SRP: Scaling and root planing
LANAP®: Laser-assisted new attachment procedure
Nd:YAG: Neodymium-doped Yttrium Aluminum Garnet
CAL: Clinical attachment level
PPD: Probing pocket depth
BOP: Bleeding on probing
*P. intermedia*: *Prevotella intermedia*
*T. denticola*: *Treponema denticola*
qPCR: Quantitative Real-time polymerase chain reaction
ENAP®: Excisional New Attachment Procedure
TBC: Total bacterial count
*A. actinomycetemcomitans*: *Aggregatibacter actinomycetemcomitans*
*P. gingivalis*: *Porphyromonas gingivalis*
BI: Bleeding index
GI: Gingival index
