# Supplementary material for: 1064 nm Nd:YAG versus 940 nm diode laser-assisted periodontal therapy in stage II periodontitis: a randomized controlled trial
Source: BMC Oral Health. 2026 Jun 1;26:1205. doi: 10.1186/s12903-026-08676-x (PMC13339547; doi:10.1186/s12903-026-08676-x)
Supplement: Supplementary file 1 — Supplementary Material 1: Table 1. 1064 nm Nd:YAG and 940 nm diode laser parameters. [file 12903_2026_8676_MOESM1_ESM.docx]

| **Laser delivery parameter** | **Nd:YAG 1^st^ pass** | **Nd:YAG 2^nd^ pass** | **Diode 1^st^ pass** | **Diode 2^nd^ pass** |
| --- | --- | --- | --- | --- |
| **Device** | **Fotona d.o.o., Ljubljana, Slovenia** | | **BIOLASE Inc., Foothill Ranch, CA, USA** | |
| **Laser emission settings** | | | | |
| **Wavelength (nm)** | 1064 | 1064 | 940 | 940 |
| **Emission Mode** | Pulsed (MSP) | Pulsed (VLP) | CW | CW |
| **Average power (W)** | 3 | 4 | 1 | 0.5 |
| **Frequency (Hz)** | 20 | 20 | NA | NA |
| **Pulse duration (µs)** | 100 | 600 | NA | NA |
| **Pulse energy (mJ/pulse)** | 150 | 200 | NA | NA |
| **Peak power (W)** | 1500 | 333 | NA | NA |
| **Applicator characteristic** | | | | |
| **Tip type and diameter (µm)** | 320  Fiber optic | | 300  Fiber optic | |
| **Laser application details** | | | | |
| **Contact mode** | Contact | Contact | Contact | Contact |
| **Direction of movement** | Coronal-apical | Apical-coronal | Coronal-apical | Apical-coronal |
| **Power density (W/cm^2^)** | 3730 | 4974 | 1415 | 707 |
| **Treatment details** | | | | |
| **Irradiation time per pocket (sec)** | ~ 20 | ~ 5 | ~ 20 | ~ 5 |
| **Purpose** | Troughing  (photothermal ablation of the pocket lining) | Blood clot formation | Troughing  (ablation of the pocket lining) | Blood clot formation |
| **No. of treatment** | 1 | 1 | 1 | 1 |

**Supplementary Table 1: 1064 nm Nd:YAG and 940 nm Diode Laser parameters**

**MSP:** micro short-pulse; **VLP:** very-long-pulse; **CW:** continuous wave; **NA:** not applicable.
